# Case of Dislocation of the Crystalline Lens from a Blow

**Published:** 1850-03

**Authors:** Francis West

**Affiliations:** Philadelphia


*Case of Dislocation of the Crystalline Lens from a blow.* By Francis West, M. D., Philadelphia.

Capt. C., of middle age, and healthy, whilst playing at cards on board of his vessel, was struck in the eye with the lighted end of a candle, thrown at him across the table. The blow, which was not at all seriously designed, was received about midnight, and on the following morning, at breakfast time, I was sent for to see him. Entire loss of vision had occurred immediately after the accident, accompanied with intense pain in the affected eye, which continued unabated at the time of my visit. There was no external mark of injury to the eye, but, on examination, the conjunctiva was found to be considerably inflamed. By considerably diminishing the light, and then presenting objects of some magnitude obliquely to the eye, it was found that the organ still retained some power of vision, but the effort to look at anything sensibly increased the pain and uneasiness. The cornea was perfectly transparent, and permitted a distinct view through the pupil of the condition of the parts within. With the exception of a very small semi-lunated portion of its superior external circumference, which remained clear and black; the pupillary opening presented a thick, milky, pearl-looking and flocculent appearance, and at points there were observable small spots of a dense chalky whiteness, caused by floating shreds of the capsule of the crystalline lens, which had become opaque. There was no observable effusion of blood in the eye. The iris, towards the inferior and internal corner of the eye, presented a very marked protrusion, occasioned evidently by the strong pressure of the upper edge of the lens, which, after having escaped through its lacerated capsule, was first anteverted, and then thrust downwards and inwards into the posterior chamber of the aqueous humor, against the iris. The sensibility of this delicate septum did not appear to be much impaired, its motions being only perceptibly diminished towards the internal canthus, by reason of the mechanical pressure which the displaced lens there exerted upon the iris. The pupil, in consequence of this impeded and irregular action of the iris, assumed a distinctly ovular shape, with the greatest diameter transversely. The crystalline lens was, apparently, so tightly impacted in its unnatural position, that the slight manipulations which it was thought proper, at the time, to practise upon the globe, exerted no influence whatever towards its restoration. It was deemed advisable, therefore, after some very gentle efforts, to test the firmness of its lodgment, to withhold any other treatment than such as was required to allay the pain, subdue the conjunctivitis, and prevent, if possible, the occurrence of more deeply seated inflammation. With this view, perfect rest was enjoined, with constant application of cold to the eye, and occasional bathing with an aqueous solution of opium. These means were successful in reducing the inflammation present, and quieting the pain which the patient continued to suffer. In a few days after the accident, all the urgent symptoms had passed away, and the pupil, throughout the greater part of its extent, had become much clearer in consequence of the absorption of the capsular shreds. The lens, however, still remained in its false position, about four-fifths of it being lodged deeply in the posterior chamber of the aqueous humour. The sight underwent very little, if any, change or improvement during the time of my attendance upon him, and at the end of a week he returned home in his vessel. This short period did not, of course, admit the expectation of any decided alteration in the condition of the crystalline lens, which could be effected only, as was supposed, through the agency of absorption.

I received a letter from my patient's friends in the course of about a month after he left Philadelphia, up to which time he stated that no further unpleasant symptoms had occurred, and that he thought he was regaining slightly the power of vision.

It is matter of much regret that the result of this case could not have been positively ascertained, as the want of such knowledge materially diminishes its interest and utility.

In regard to the exact mode by which the blow from the candle produced the injury to the eye, it may be questioned whether it was by the direct impulsion of the foreign body upon the globe of the eye, or by the sudden and violent contraction of the muscles of the lids, induced by the natural and instinctive disposition of the eye to protect itself from the contact of external substances. We incline to the belief that the latter is the more satisfactory explanation of the phenomenon.

In regard to this instinctive vigilance of the eye-lids, it is well known that, now and then, it is eluded by foreign substances, which thus gain access within their enclosure. A curious instance of the kind recently came under my notice. A jeweller, who was melting some solder, imprudently poured the fluid, whilst boiling, from the original vessel into another one, which happened to contain some water; an explosion at once occurred, and a considerable portion of the melted solder found its way into one of the poor fellow's eyes. I saw him in a few moments afterwards, and on separating the lids, I discovered that the space between the lower one and the globe was completely occupied by a solid coat of the metal, which was at once removed by the forceps. It presented exactly the mould of the lower section of the eye, being semi-lunated in form, and about one fourth of an inch in diameter; in thickness it was a line or more.

Mackensie and other writers upon the diseases of the eye, have related several instances of dislocation of the crystalline lens similar to the above, but according to the maxim "observationes addendæ sunt," I thought it might be worth while also to place the present one upon record.

